# Changes in expression of the long non-coding RNA *FMR4* associate with altered gene expression during differentiation of human neural precursor cells

**DOI:** 10.3389/fgene.2015.00263

**Published:** 2015-08-10

**Authors:** Veronica J. Peschansky, Chiara Pastori, Zane Zeier, Dario Motti, Katya Wentzel, Dmitry Velmeshev, Marco Magistri, John L. Bixby, Vance P. Lemmon, José P. Silva, Claes Wahlestedt

**Affiliations:** ^1^Center for Therapeutic Innovation and Department of Psychiatry and Behavioral Sciences, Miller School of Medicine, University of MiamiMiami, FL, USA; ^2^Miami Project to Cure Paralysis, University of MiamiMiami, FL, USA; ^3^Department of Neurological Surgery, Miller School of Medicine, University of MiamiMiami, FL, USA; ^4^Center for Computational Science, University of Miami, Coral Gables, FLUSA; ^5^Department of Molecular and Cellular Pharmacology, Miller School of Medicine, University of MiamiMiami, FL, USA

**Keywords:** lncRNA, intellectual disability, epigenetics, differentiation, chromatin remodeling, *MBD4*, *FMR4*, Fragile X

## Abstract

CGG repeat expansions in the Fragile X mental retardation 1 (*FMR1*) gene are responsible for a family of associated disorders characterized by either intellectual disability and autism Fragile X Syndrome (FXS), or adult-onset neurodegeneration Fragile X-associated Tremor/Ataxia Syndrome. However, the *FMR1* locus is complex and encodes several long non-coding RNAs, whose expression is altered by repeat expansion mutations. The role of these lncRNAs is thus far unknown; therefore we investigated the functionality of *FMR4*, which we previously identified. “Full”-length expansions of the *FMR1* triplet repeat cause silencing of both *FMR1* and *FMR4*, thus we are interested in potential loss-of-function that may add to phenotypic manifestation of FXS. Since the two transcripts do not exhibit *cis*-regulation of one another, we examined the potential for *FMR4* to regulate target genes at distal genomic loci using gene expression microarrays. We identified *FMR4*-responsive genes, including the methyl-CpG-binding domain protein 4 (*MBD4*). Furthermore, we found that in differentiating human neural precursor cells, *FMR4* expression is developmentally regulated in opposition to expression of both *FMR1* (which is expected to share a bidirectional promoter with *FMR4*) and *MBD4*. We therefore propose that *FMR4*’s function is as a gene-regulatory lncRNA and that this transcript may function in normal development. Closer examination of *FMR4* increases our understanding of the role of regulatory lncRNA and the consequences of *FMR1* repeat expansions.

## Introduction

Fragile X Syndrome, FXTAS, and FXPOI are X-linked disorders that arise from expansions in a CGG-repeat region in the 5′-UTR of the *FMR1* gene. Normal *FMR1* alleles contain 6–54 repeats, expansions from 55 to 200 repeats are considered “premutations” and all larger repeat sizes are known as the “full mutations.” Individuals with a premutation may develop the adult-onset neurodegenerative disorder known as FXTAS, while women carrying the premutation are at risk for FXPOI. Only the full mutation leads to FXS, which is a common cause of inherited intellectual disability and autism (Oostra and Willemsen, 2003). *FMR1* premutations result in overproduction of toxic, expanded mRNAs that contribute to the development of FXPOI and FXTAS pathology (Tassone et al., 2000; Kenneson et al., 2001). Full mutations lead to DNA and repressive histone methylation of the *FMR1* locus (Sutcliffe et al., 1992; Hornstra et al., 1993; Coffee et al., 1999, 2002; Kumari and Usdin, 2010). Thus FXS derives from the loss of *FMR1* mRNA and protein FMRP. We and others have identified four non-coding transcripts with abnormal expression in response to Fragile X repeat expansions at the *FMR1* locus (Ladd et al., 2007; Khalil et al., 2008; Pastori et al., 2014), but their role in FXS/FXTAS/FXPOI phenotypes remains to be determined.

The vast majority of the human transcriptome is comprised of either long [>200 nucleotides (nt)] or short ncRNAs (Cheng et al., 2005; Banfai et al., 2012; Djebali et al., 2012). While short ncRNAs typically regulate gene expression through posttranscriptional mechanisms or by interfering with translation (Rother and Meister, 2011; Fabian and Sonenberg, 2012), lncRNAs (which can be many kilobases long) often act in *cis* or *trans* to regulate gene expression at their locus of origin or elsewhere in the genome, respectively. Evidence suggests that lncRNAs perform scaffolding functions by recruiting epigenetic complexes or ribonucleoproteins that cause chromatin remodeling (Wang and Chang, 2011). Other lncRNAs act post-transcriptionally by targeting mRNAs or translational machinery. Regardless of the mechanism, a growing body of evidence implicates lncRNAs in a myriad of normal cellular functions such as DNA damage response and mitosis (Tsai et al., 2010; Yap et al., 2010; Hung et al., 2011; Kotake et al., 2011; Wang and Chang, 2011) and in diseases, such as cancer (Hajjari et al., 2014).

Recent attention has focused more specifically on the role of lncRNAs in neurodevelopmental programs and diseases of the nervous system. For example, lncRNAs are involved in the differentiation of neural cell types, and synaptic signaling and maturation (Mercer et al., 2010; Qureshi et al., 2010). In addition, both short and long ncRNAs are known to be involved in Prader–Willi syndrome, which is a developmental disorder caused by paternal deletion of a maternally imprinted region and can present with metabolic dysregulation including circadian rhythm defects (Sahoo et al., 2008; De Smith et al., 2009; Powell et al., 2013). Both syndromic and non-syndromic ASD susceptibility loci also contain aberrantly expressed lncRNAs that may contribute to disease (Velmeshev et al., 2013; Ziats and Rennert, 2013). Dysfunction of lncRNAs has also been linked to pathogenesis of neurodegenerative disorders including Alzheimer’s disease (Faghihi et al., 2008) and spinocerebellar ataxia type 7, another repeat expansion disorder (Sopher et al., 2011). In sum, there is a growing body of evidence supporting the involvement lncRNAs in both the normal and diseased nervous system, spurring further mechanistic inquiries.

*FMR4*, an untranslated, antisense lncRNA originating at the *FMR1* gene locus was shown to have anti-apoptotic functions in HEK293T and HeLa cells but to have no effect on expression of *FMR1* (Khalil et al., 2008). Here, we describe *FMR4*’s function as a regulator of gene expression in *trans* by identifying mRNA expression changes induced by *FMR4*. In particular, these effects in HEK293T cells are mirrored by discordant developmental regulation between *FMR4* and one of its targets, *MBD4*, in hNPCs.

## Materials and Methods

### HEK293T Cell Culture, Transfection, and RNA Extraction

HEK293T cells were cultured in DMEM with 10% FBS. In overexpression experiments, cells were transfected with pcDNA3.1-FMR4 or the empty pcDNA3.1 control vector using Lipfectamine 2000CD. For knockdown experiments, the siRNA FMR4(C) (Khalil et al., 2008), versus Silencer Negative Control siRNA #1 (Ambion) were used with the Lipofectamine RNAiMAX transfection reagent, according to manufacturer’s instructions (Invitrogen). For microarray experiments, 1 × 10^6^ cells were plated and transfected with 0.5 μg plasmid or 40 nM siRNA on the same day, and incubated for 6 or 24 h after transfection. For validation, *FMR4* was knocked down using two sequential siRNA transfections over 72 h. RNA was extracted using Trizol (Invitrogen) and the RNeasy Mini Kit, and treated with DNAse on-column using the RNAse-free DNAse Set (Qiagen) according to manufacturer’s instructions.

### Microarray Hybridization and Analysis

At 6 or 24 h post transfection, RNA was extracted and samples were submitted to the Hussman Institute for Human Genomics Center for Genome Technology for microarray analysis using Affymetrix Human Gene 2.0 ST Arrays. Total RNA samples were first prepared using the Ambion WT Expression Kit (cat# 4411974). Briefly, the kit generates sense-strand cDNA from total RNA using a reverse transcription priming method that specifically primes non-ribosomal RNA, including both poly(A) and non-poly(A) mRNA. Next, samples are fragmented and labeled using the Affymetrix GeneChip WT Terminal Labeling Kit (cat# 902280). Final yield was hybridized onto the array, washed and stained using the Affymetrix GeneChip Hybridization, Wash, and Stain Kit (cat# 900720). Arrays were scanned using GeneChip Scanner 3000 7G system. Background subtraction, GC-RMA normalization and quality control were performed using the Affymetrix GeneChip Command Console Software and the bioconductor package from R. Data have been archived in the Gene Expression Omnibus at the National Center for Biotechnology Information, and assigned the accession number GSE70817.

### cDNA Synthesis and Quantitative Real-Time PCR (qPCR)

cDNA was synthesized using the High Capacity cDNA Reverse Transcription Kit (Applied Biosystems) with 500 ng of total RNA per reaction. Gene-specific FMR4 cDNA was primed separately (“FMR4 RT”: ATTGCTGGCAGTCGTTTCTT), in order to specifically detect the antisense transcript and prevent capture of overlapping sense transcripts arising from that genomic region. Random hexamer-primed cDNA libraries were used for detection of all other genes. FMR4 RNA expression was quantified using SYBR Green quantitative real-time PCR (qPCR) with the following primers, validated by melting curve: “FMR4 FW” – ACCAAACCAAACCAAACCAA and “FMR4 REV” – GTGGGAAATCAAATGCATCC. Commercially available TaqMan probes (Invitrogen) were used for all other transcripts (MBD4, cat# Hs00187498_m1; FMR1, cat# Hs00924547_m1; MALAT1, cat# Hs01910177_s1). The endogenous control was glyceraldehyde 3-phosphate dehydrogenase (GAPDH, cat# 4326317E) where necessary, and data were analyzed by the ΔΔCt method. For polyA detection, cDNA was synthesized from 1 μg total RNA using the High Capacity cDNA kit and oligodT primers at 50 nM final concentration. In the noRT control, we omitted the reverse-transcriptase enzyme from the cDNA synthesis reaction. Reverse transcription products were amplified using qPCR as described above and visualized on a 1.5% agarose gel.

### Human Neural Precursor Cell (hNPC) Culture, Differentiation, and Viral Transduction

hNPCs used in this study were derived from human fetal brains collected from third trimester aborted fetuses received from the Birth Defects Research Lab at the University of Washington in Seattle. This work was classified as “Non-Human Subject Research” by the Human Subject Research Office at the University of Miami, and therefore was not subject to Institutional Review Board approval. Briefly, brain tissues were dissected and dissociated using the trypsin-based Neural Tissue Dissociation Kit (Miltenyi Biotec, cat #130-093-231). Five million single cells were seeded into each 75-mm tissue culture flask in proliferation media supplemented with B27 (1X of proprietary formula), human Epidermal Growth Factor (hEGF) at 20 ng/mL, human Fibroblast Growth Factor (hFGF) at 10 ng/mL, heparin at 20 ug/mL, GlutaMax (1X, Gibco), and Primocin at 0.1 mg/mL (InvivoGen). Neural stem cells formed neurosphere colonies after approximately 7 DIV, while other cells remained in suspension as single cells or formed a monolayer on the flask surface. Neurospheres can be maintained as such in suspension for several months using proliferation media, or hNPCs can be differentiated, forming a mixture of neurons and astrocytes. Neurospheres were transduced with a lentiviral vector expressing *FMR4* (pLentiCMV/TO-mCherry-FMR4) or the control vector (pLentiCMV/TO-mCherry), and collected for qPCR analysis after 2 days. To differentiate, neurospheres are dissociated into single cells with Accutase and cultured in Advanced DMEM/F12 media without hFGF or hEGF, but with Bottenstein’s N2 at 1X (Invitrogen, proprietary formula), 2.5% fetal bovine serum, heparin at 20 μg/mL, GlutaMax (1X, Gibco) and Primocin at 0.1 mg/mL (InvivoGen).

### Subcellular Fractionation

Neurospheres were collected by centrifugation at 250 × *g* in order to form 50 μL pellets. Pellets were washed with PBS and fractionated with the NE-PER Nuclear and Cytoplasmic Extraction Reagents (Thermo Scientific, cat# 78835) according to manufacturer’s instructions. Briefly, cells were sequentially lysed and centrifuged to first separate pelleted nuclei from cytoplasm, then to separate chromatin from nucleoplasmic components. RNA was extracted from solid chromatin with 1 mL of Trizol, and with 0.75 mL Trizol LS for every 0.25 mL of liquid fraction. cDNA prepared using random primers was used for TaqMan qPCR (*MALAT1*, *GAPDH*, and *FMR1*), while gene-specific priming and SYBR Green was used for *FMR4* as noted above. In each case, starting material for cDNA reactions was 2 μL total RNA (not equal mass of RNA), to enable comparison of absolute quantities of each transcript between compartments. Relative quantification (RQ) for each transcript in each compartment was calculated from Cq values by qPCR. Individual fraction RQ values were normalized to the total detected amount for each transcript.

## Results

### *Fmr4* Induces Genome-Wide Changes in Gene Expression

**FIGURE 1:**
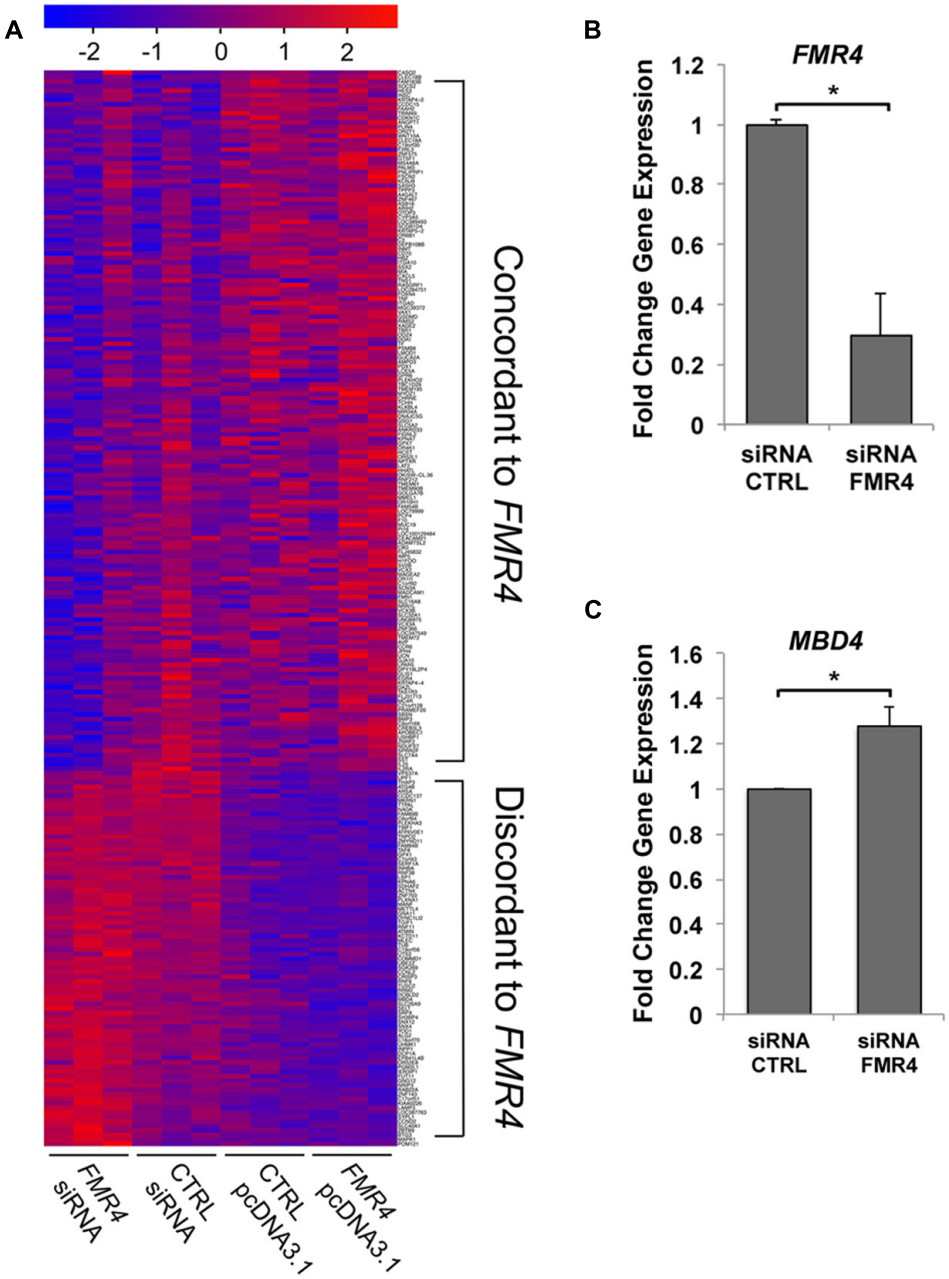
**Microarray analysis of mRNA expression changes in response to *FMR4* knockdown and overexpression reveal *trans*-regulatory targets. (A)** Microarray analysis of HEK293T cells with *FMR4* overexpression or knockdown (*n* = 3). LIMMA analysis and Cluster Affinity identified genes concordantly or discordantly regulated compared to *FMR4*. **(B)** *FMR4* was knocked down with siRNA in HEK293T cells in order to validate changes in a putative *FMR4*-sensitive gene identified by microarray **(C)**. (*n* = 6, *p < 0.05 by Student’s *t*-test).

Previous studies of the 2.4 kb antisense lncRNA *FMR4* described no *cis-*regulation of *FMR1* (Khalil et al., 2008); therefore we hypothesized that *FMR4* would regulate gene expression in *trans*, which is a well-documented function of other lncRNAs (Rinn et al., 2007; Kino et al., 2010; Miyagawa et al., 2012). In order to comprehensively measure gene regulation in response to *FMR4* at the mRNA level, we treated HEK293T cells with either an siRNA against *FMR4*, a scrambled control siRNA (knockdown), pcDNA3.1-*FMR4*, or the empty pcDNA3.1 vector (overexpression) and processed for microarray hybridization after 6 or 24 h. Using LIMMA, a linear modeling approach (Smyth, 2004), we identified differential expression of over 3,700 genes between *FMR4* knockdown, overexpression and their respective control conditions, and characterized the pattern of target gene expression relative to *FMR4*. To this end, we used the Cluster Affinity tool of TIGR MultiExperiment Viewer to identify genes with opposite behavior in the knockdown condition compared to the overexpression condition. This strategy narrowed our focus to the 238 transcripts represented in **Figure 1A** and Supplementary Figure S1, which are further classified by whether they are concordant or discordant with respect to *FMR4* changes. This analysis yielded 155 and 83 target genes with concordant and discordant changes in mRNA expression relative to *FMR4*, respectively. These data support the idea that *FMR4* is a regulator of gene expression through *trans*-activity.

### Pathway Analysis and *FMR4* Target Validation

We then analyzed the cohort of concordant and discordant *FMR4*-responsive genes with GeneGo Metacore (**Tables 1** and **2**). Cell cycle regulation and apoptosis were highly ranked biological processes affected by *FMR4*, which is consistent with our earlier findings (Khalil et al., 2008). Additionally, *FMR4*-sensitive genes are enriched in developmental processes in general and neurodevelopmental processes in particular (e.g., adrenergic and opioid signaling, Wnt pathway, cytoskeletal elements, synaptogenesis). Informed by the pathway analysis and previous insights into *FMR4*’s function, we used HEK293T cells with *FMR4* knocked down by sequential siRNA transfections (**Figure 1B**) to validate a 28% increase in methyl CpG-binding domain (*MBD4*; **Figure 1C**).

**Table 1:** Top GeneGo pathways represented by *FMR4*-responsive genes identified by microarray analysis.

Category	GeneGo pathway	*p*-value
Cell adhesion	Chemokines and adhesion	0.10161
	Integrin-mediated cell adhesion and migration	0.11458
Cell cycle	Regulation of G1/S transition (part 2)	0.00000
	Nucleocytoplasmic transport of CDK/Cyclins	0.03480
	Spindle assembly and chromosome separation	0.08019
Development	Mu-type opioid receptor signaling via beta-arrestin	0.00000
	S1P1 receptor signaling via beta-arrestin	0.00000
	Beta-adrenergic receptors transactivation of EGFR	0.00000
	Ligand-independent activation of ESR1 and ESR2	0.00001
	Gastrin in cell growth and proliferation	0.00002
	Alpha-2 adrenergic receptor activation of ERK	0.00002
	Regulation of epithelial-to-mesenchymal transition (EMT)	0.04670
	Lipoxin inhibitory action on PDGF, EGF, and LTD4 signaling	0.08717
	A3 receptor signaling	0.11683
Immune response	Oncostatin M signaling via MAPK in mouse cells	0.00000
	Oncostatin M signaling via MAPK in human cells	0.00000
	IL-15 signaling	0.00002
	Histamine H1 receptor signaling in immune response	0.02758
Transport	RAN regulation pathway	0.04453
N/A	Inhibitory action of Lipoxin A4 on PDGF, EGF, and LTD4 signaling	0.08485

**Table 2:** Top GeneGo process networks represented by *FMR4*-responsive genes identified by microarray analysis.

Category	GeneGo process networks	*p*-value
Apoptosis	Death domain receptors and caspases in apoptosis	0.02682
Cell adhesion	Platelet aggregation	0.02999
	Leucocyte chemotaxis	0.04076
Cell cycle	G1/S Interleukin regulation	0.00000
	G1-S Growth factor regulation	0.00002
Cytoskeleton	Actin filaments	0.00022
	Actin filaments	0.03088
Development	Hemopoiesis, erythropoietin pathway	0.00000
	Neurogenesis and synaptogenesis	0.08499
	Neuromuscular junction	0.11290
	Wnt/beta-catenin, notch, VEGF, IP3, and integrin signaling	0.11674
DNA Damage	Checkpoint	0.00006
Immune response	BCR pathway	0.00008
Inflammation	MIF signaling	0.00000
	IL-2 signaling	0.00003
	Protein C signaling	0.06668
	Kallikrein–kinin system	0.09179
Reproduction	FSH-beta signaling pathway	0.00001
Signal	CREM pathway	0.00002
Transduction	Neuropeptide signaling pathways	0.01407

### Developmental Regulation of Gene Expression by *FMR4* in hNPCs

To investigate the putative role of *FMR4* in neurodevelopment and Fragile X-associated neurological disorders, we used an *in vitro* model system consisting of human fetal-derived neurospheres (hNSs). These cells can be maintained as precursor in hNSs, or induced to differentiate into a mixed culture of early neurons and glia (see Materials and Methods). This system has the advantage of being a primary culture of human brain cells (critical for studying a primate-specific transcript *in vitro*) without the need for reprogramming, as would be the case with embryonic or induced pluripotent stem cells. We focused on the relationship between *FMR4* and its putative target gene, *MBD4*, which we identified by microarray analysis. *MBD4* is a transcriptional repressor involved in DNA repair (Ballestar and Wolffe, 2001; Kondo et al., 2005). Expression of *MBD4* developmentally regulates several tissues (Ruddock-D’cruz et al., 2008; Zhang et al., 2014a), and its aberrant expression in hippocampal GABAergic neurons in psychiatric disease may be linked to abnormal differentiation in these cells (Benes et al., 2009).

**FIGURE 2:**
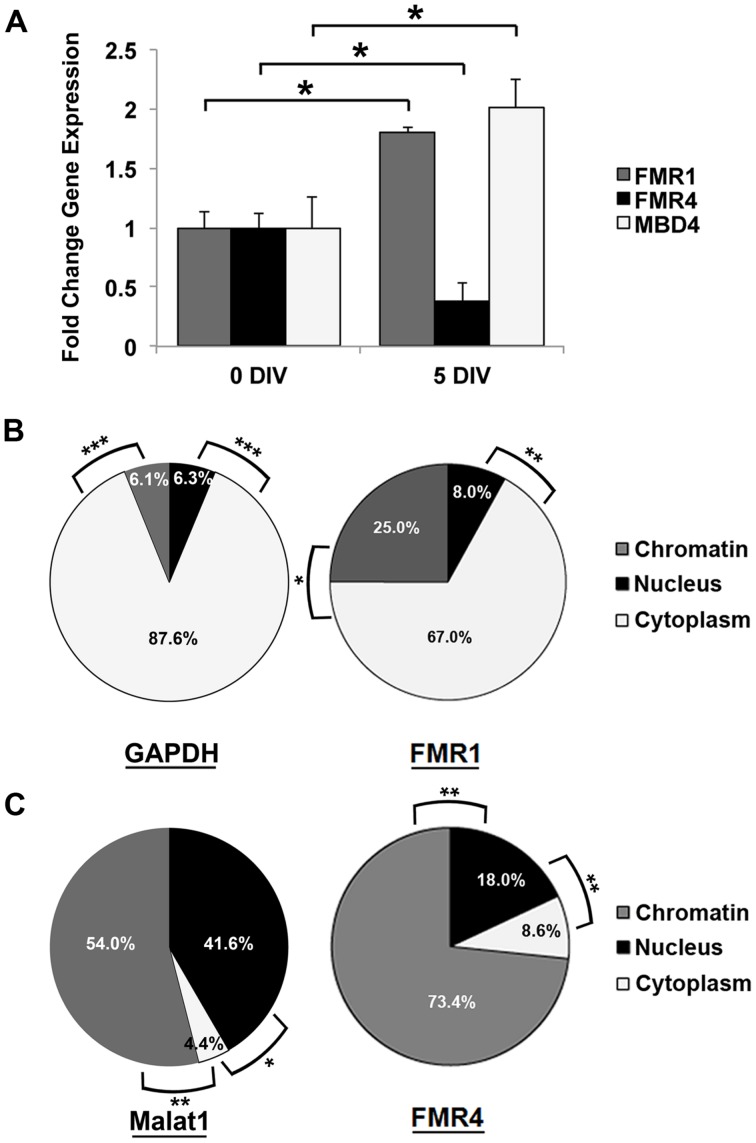
**Expression of *FMR4* and its target *MBD4* during differentiation of hNPCs may be related to chromatin-modulatory function of the lncRNA. (A)** Comparison of RNA expression between proliferating neurospheres (0 DIV) and differentiating hNPCs (5 DIV) revealed increased *FMR1* and decreased *FMR4* after 5 days of differentiation (*n* = 5, *p < 0.05 by Student’s *t*-test). As predicted, the putative *FMR4* target gene *MBD4* was upregulated with decreased *FMR4*. Subcellular fractionation of hNPCs with subsequent qPCR detection confirmed the cytoplasmic localization of mRNAs such as **(B)** *GAPDH* and *FMR1*. *MALAT1* **(C)**, known to be associated to nuclear speckles, was significantly enriched in the nucleus and chromatin compared to cytoplasm. *FMR4* RNA **(C)** was largely localized to chromatin. (*n* = 5, *p < 0.05, **p < 0.01, ***p < 0.001 by One-way ANOVA with Tukey HSD).

The *FMR1* locus is crucial to normal brain development; thus, we wanted to determine whether *FMR4* expression is dependent on developmental stage. We extracted RNA from undissociated hNS [“0 days *in vitro*” (DIV)] and cultured cells from dissociated hNS up to five DIV in differentiation media. At both time points, we measured *FMR4* expression as well as that of *FMR1*, to determine whether these transcripts are independently regulated. We observed that *FMR4* expression is significantly decreased in differentiating cells at 5 DIV while *FMR1* is increased at the same time point (**Figure 2A**). We then measured *MBD4* and found that it is also developmentally regulated. As indicated by the microarray analysis, *MBD4* was increased at a time point when *FMR4* expression is low (**Figure 2A**). We also observed changes in expression level of other *FMR4* target genes in undifferentiated, proliferating hNPCs transduced with an mCherry-tagged lentiviral vector expressing *FMR4* (Supplementary Figure S2A). We found that *FMR4* overexpression significantly upregulated two putative targets, the deubiquitinase *YOD1* and the G-protein subunit *GNG12*, and downregulated the ribonucleotide reductase *RRM2* (Supplementary Figure S2B). These data corroborate our finding that *FMR4* regulates gene expression in *trans*.

### Molecular Mechanisms of *FMR4*

To better understand the molecular role of *FMR4*, we performed subcellular fractionation of hNS. As expected, *GAPDH* and *FMR1* mRNA were highest in the cytoplasmic fraction (87.6 and 67.0%, respectively) where they are translated (Duszczyk et al., 2011) (**Figure 2B**). Some lncRNAs are expressed predominantly in the nucleus. A well-known example of this is the lncRNA *NEAT2/MALAT1* (Gutschner et al., 2013). We measured this transcript as a positive control for nuclear transcripts, and found it enriched in the nucleoplasm (41.6%) and chromatin (54.0%) relative to the cytoplasm (**Figure 2C**). *FMR4* RNA was primarily localized (73.4%) to chromatin (**Figure 2C**), which is consistent with a transcriptional regulatory function of *FMR4*.

Since RNA polyadenylation frequently targets mature RNA for export into the cytoplasm (Zhang et al., 2014b); many lncRNAs are not polyadenylated. Therefore, to establish whether *FMR4* can be polyadenylated, we performed first-strand cDNA synthesis with oligodT primers, thereby capturing polyadenylated transcripts from total hNS RNA. We observed *via* qPCR that in addition to *FMR1* and *GAPDH* mRNAs (which are expected to be polyadenylated), a detectable portion of *FMR4* is also polyadenylated (Supplementary Figure S3). These data suggest that a fraction of *FMR4* may be stabilized by polyadenylation; this would increase the molecule’s half-life and permit its diffusion to distant genomic loci.

## Discussion

Loss of *FMR1* mRNA and FMRP function has been widely studied, but there is relatively little known about ncRNA encoded by the locus. Here we show that one such ncRNA, *FMR4*, may regulate mRNA expression genome-wide *via* a developmentally regulated transcriptional mechanism, thereby impacting important biological processes.

Similar to *FMR4*, *trans*-acting lncRNAs [such as *HOTAIR*, *MALAT1*, and *GAS5* (Rinn et al., 2007; Kino et al., 2010; Miyagawa et al., 2012)] affect loci far from their genomic locus of origin. In this study, we confirmed that knockdown and overexpression of *FMR4* causes changes in genes involved in proliferation and differentiation. As a chromatin-associated transcript, *FMR4* may act at the transcriptional level by forming complexes with histone modifying enzymes or by directly targeting mRNA stability, splicing, or editing. It remains to be seen whether these observations are dependent on RNA-protein interactions, and whether they result from direct epigenetic changes or *via* downstream effects.

Our data show that *FMR4* is developmentally regulated in an hNPC model. After 5 days of *in vitro* differentiation, *FMR4* expression is significantly reduced, while that of both *FMR1* and the *FMR4* target gene *MBD4* is increased. Since *FMR4* and *FMR1* do not interact in *cis* but are discordantly expressed with differentiation of hNPCs, the bidirectional promoter responsible for expression of both of these transcripts might be activated in only one direction at a time. An alternative possibility is that *FMR4* RNA is degraded at a higher rate during this period while *FMR1* is not. Nevertheless, it would be interesting to determine whether transcription factors normally regulate this locus as a whole or target each transcript individually. It is also unclear whether the decrease in *FMR4* lncRNA expression contributes to disease in addition to loss of *FMR1*, or is an artifact of the locus-wide, full mutation-induced epigenetic changes causing transcriptional repression. Future studies will be necessary to distinguish between these possible mechanisms and consequences of *FMR4* regulation.

Discordant regulation of *MBD4* and *FMR4* leads us to conclude that *FMR4* regulates expression of genes at distal loci, as the same relationship was identified by our overexpression and knockdown studies. Our independent validation studies confirm this was not due to false positive identification, which is a common problem in genome-wide analyses such as microarray studies. *FMR4*’s localization to the chromatin fraction points to a transcriptional mechanism for this effect, however, studies evaluating the direct binding of *FMR4* to nucleic acids or proteins are warranted. Such studies could suggest a direct role for *FMR4* in specifically regulating *MBD4* and gene expression more broadly, although this cannot be concluded definitively based solely on changes in mRNA levels. We acknowledge that differences in RNA processing or any number of effects downstream of *FMR4* could be responsible for the observed differential gene expression, therefore it would be useful to examine precise interactions between *FMR4* and its targets. Chromatin immunoprecipitation experiments could, for example, establish an interaction between *FMR4* and promoter regions of target genes. Based on the function of other lncRNAs, one could speculate that *FMR4* helps form three-dimensional interactions between distant sequence elements such as promoters and enhancers (Ma et al., 2014), and that *FMR4* polyadenylation targets this transcript for further RNA processing (Norbury, 2013). Answering these questions would help our understanding of *FMR4* function in particular and continue the rapid expansion of evidence on the elements that govern lncRNA actions in general.

In this study we have described novel facets of *FMR4* functionality as it relates to neurodevelopment, and suggest that perturbation in the expression of this lncRNA may contribute to pathogenesis of the Fragile X repeat expansion-associated disorders. We report that *trans-*regulatory activity of *FMR4* is corroborated by changes in target gene expression during differentiation of hNPCs. With this new information, we have further developed the evidence supporting the role of primate-specific lncRNAs in complex developmental programs and opened new avenues of research into the causes and therapies for Fragile X-associated neurological disorders.

## Author Contributions

VP, CP, DM, KW, DV, and MM contributed to the acquisition, analysis, and interpretation of data. VP, CP, ZZ, JB, VL, JS, and CW made substantial contributions to the conception and design of the work. All authors participated in drafting and/or revising the manuscript for intellectual content, approval for publication and agree to be accountable for all aspects of the work.

## Conflict of Interest Statement

The reviewer Guney Bademci declares that, despite being affiliated with the same institute as the authors Veronica J. Peschansky, Chiara Pastori, Zane Zeier, Dario Motti, Katya Wentzel, Dmitry Velmeshev, Marco Magistri, John L. Bixby, Vance P. Lemmon, José P. Silva, and Claes Wahlestedt, the review process was conducted objectively.

## Abbreviations

ASD: autism spectrum disorder
DIV: days *in vitro*
FMRP: Fragile X mental retardation protein
FXPOI: Fragile X-related primary ovarian insufficiency
FXS: Fragile X syndrome
FXTAS: Fragile X-associated tremor/ataxia syndrome
hNPCs: human neural precursor cells
hNS: human neurospheres
lncRNAs: long non-coding RNAs
mRNA: messenger RNA
ncRNAs: non-coding RNAs
