# Supplementary material for: Changes in expression of the long non-coding RNA FMR4 associate with altered gene expression during differentiation of human neural precursor cells
Source: Front Genet. 2015 Aug 10;6:263. doi: 10.3389/fgene.2015.00263 (PMC4530595; doi:10.3389/fgene.2015.00263)

Supplementary Figure 1. List of genes with differential expression in response to both *FMR4* upregulation and down regulation, ordered as presented in main Figure 1.

Concordant to *FMR4*

CASQ2
CLEC18B
FAM183B
SOCS2
HES3
INSC
KRTAP4-2
CCDC15
FAAH2
TRIM49
CDKN1C
ANGPT1
PLIN4
OR2T1
WNT10A
CLEC18A
C19orf30
F2RL3
ZNF575
GTSF1
MS4A6A
PALM3
PNLIPRP1
FSCN2
KCNJ9
SASH3
TPPP3
A4GALT
ZNF467
ASB16
ARIH2
OTOP3
CYP3A5
LOC389493
SCGB1D4
KRTAP5-2
OR6B1
C3
DEFB108B
INMT
CD70
HBZ
ITGA10
SSX2
MIA
CXCL5
TNS1
RASGRF1
LOC284751
FOXN4
TNF
ITGAD
MGC39372
VAX1
GSDMD
RIMS2
XAGE2
TBR1
ODZ4
DGKI
TF
PSMB8
LMOD1
GUCA2A
AMPD3
PDX1
LCE5A
GPR6
PLEKHO2
TBC1D29
TMEM195
MYOZ1
CHRNE
TCHH
KLKBL4
MIR34A
DNAJC5G
GSG1
SLC5A2
ANKRD33
FIGNL2
KPNA7
GPX7
OR4K1
HCST
OR52L1
NPTXR
LAT2
HHATL
OK/SW-CL.36
RNF212
TMEM61
TMEM90B
GOLGA7B
MMEL1
OR10H1
FAM54B
LOC79999
PCP4
F10
MUC19
PI16
LOC100129484
CEACAM21
ADAMTSL2
C8G
FLJ45832
IMP5
H1FOO
SV2B
VCX2
MAGEA2
OR1I1
C1orf92
SCN3A
MADCAM1
FMN1
SLC16A8
NRN1L
VCX3B
SLC32A1
UNQ6975
VCX3A
ZNF366
LOC347549
TMEM72
AVP
CCR6
JPH4
UCN
GJA10
LPAR5
DPY19L2P4
GLIS1
EGR4
KRTAP4-4
DAZL
TAS1R3
FLJ31713
MC4R
C21orf128
PRAMEF20
SBSN
BMP3
C9orf169
CREB3L3
APOBEC1
USHBP1
ZNRF2
NDUFS7
SPRR2F
SLC7A4
SST
IL25
IL2RA

Discordant to *FMR4*

VPS37A
UPF1
THAP3
ATG4B
ARSA
CCDC137
MKRN1
TTPAL
NAGK
FAM89B
C6orf64
PLEKHA3
TWF1
ATP6V0E1
TNPO2
ZMYND11
FAM84B
TAF8
GPX1
C7orf43
SERF1A
INHBA
RNF38
LSP1
KPNA6
SDHAF2
ACTN4
ZNF703
PLXNA1
MANF
METTL4
GNA11
DYNC1LI2
TGIF1
RNF11
ATMIN
KCTD11
MLEC
TUB
C19orf56
UTS2
COMMD1
UBE2Z
SGK269
CCNL2
CRISP3
RNF6
TUSC2
RRM2
DCBLD2
MBD4
SLC26A9
SELT
SRPX
SH3BP4
SNX12
SNX4
YOD1
ALG2
C16orf70
UHMK1
INPP1
DCP1A
EPB41L4B
OR52E8
PGM2L1
IER3IP1
FUT11
GNG12
NRIP3
RAB22A
ZNF143
C17orf51
KIAA0226
LAMP3
LOC387763
SYPL1
CCND2
SLC40A1
ZBTB9
BTG3
MAPK1
POM121

Supplementary Figure 2. Overexpression of *FMR4* in human neurospheres causes downstream gene expression changes. (A) Human neurospheres were transduced with an mCherry-tagged lentivirus resulting in *FMR4* overexpression. (B) Overexpression of FMR4 in undifferentiated hNPCs results in differential expression of the genes *GNG12*, *RRM2* and *YOD1* (n=6, *=p<0.05, **=p<0.01).

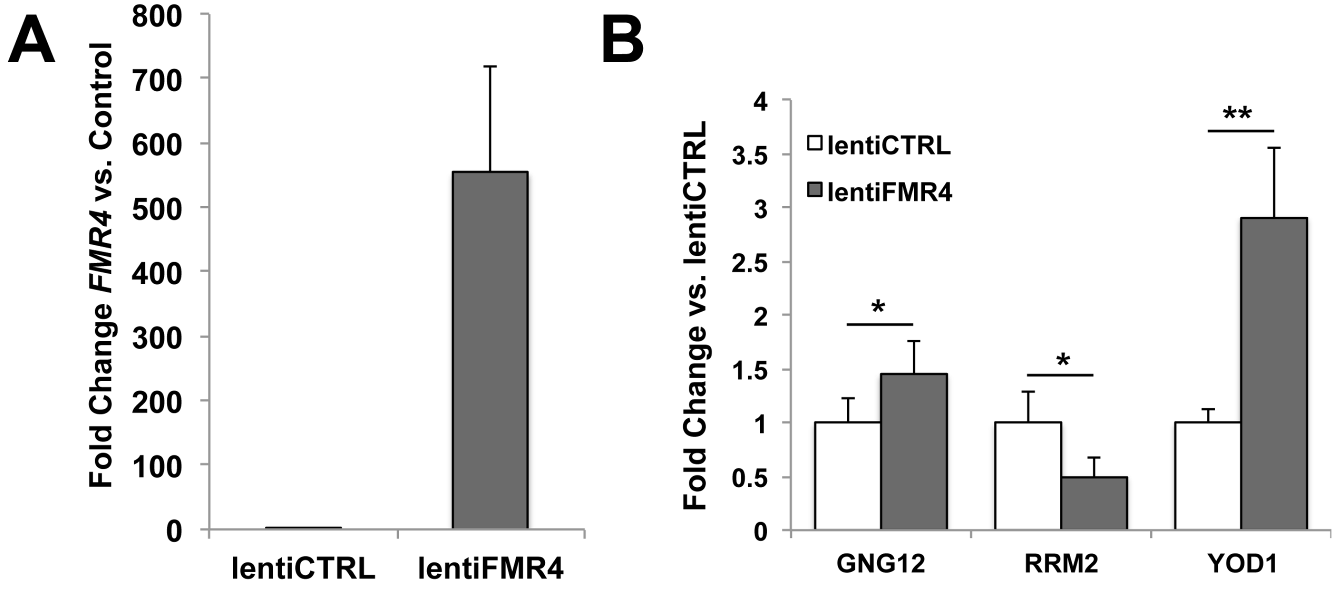

Supplementary Figure 3. FMR4 is polyadenylated. Reverse transcription of total RNA from hNPCs using oligodT primers revealed detectable levels of polyadenylated *FMR4* RNA, in addition to the polyadenylated mRNAs *FMR1* and *GAPDH*.

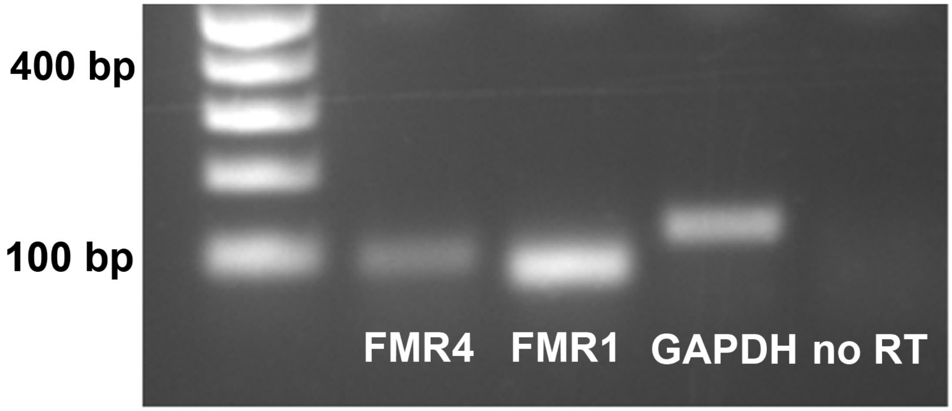

Supplement: Supplementary file 1 [file Image_1.PDF]
